# Latent Profiles of Posttraumatic Growth and Their Relation to Differences in Resilience among Only-Child-Lost People in China

**DOI:** 10.1371/journal.pone.0167398

**Published:** 2016-12-22

**Authors:** Wen Zhang, An-ni Wang, Shu-yu Yao, Yuan-hui Luo, Zhi-hua Li, Fei-fei Huang, Hui Li, Yi-zhen Yin, Jing-ping Zhang

**Affiliations:** 1 Nursing Psychology Research Center, Xiangya Nursing School, Central South University, Changsha, China; 2 Institute of Education, Hunan Agriculture University, Changsha, China; 3 School of nursing, Fujian medical university, Fuzhou, China; Harvard Medical School, UNITED STATES

## Abstract

**Aims:**

Since the early 1980s, the one-child policy has been implemented nationwide in China. A special group called the “only-child-lost family” (OCL family) has emerged and has become a social phenomenon that cannot be ignored. We report latent profiles of posttraumatic growth and their relation to differences in resilience among OCL people in China.

**Methods:**

A total of 222 OCL people were investigated using the Posttraumatic Growth Inventory and the Connor-Davidson Resilience Scale. Latent profile analysis was applied to explore PTG latent profiles. Multinomial logistic regression was used to analyze the socio-demographic variables in each latent profile and the association between profile membership and resilience.

**Results:**

Three latent profiles were identified and labeled the “high appreciation-power group” (30.6%), the “general moderate growth group” (47.7%) and the “low growth and extreme possibility group” (21.7%). Compared to those in the high appreciation-power group, individuals with monthly income >2000 ($312) were less likely to be in the general moderate growth group (OR = 0.13, *P*<0.01), whereas individuals with a spouse were less likely to be in the low growth and extreme possibility group (OR = 0.43, *P*<0.01). Individuals in the “general moderate growth group”(OR = 0.92, *P*<0.01, 95%CI:0.89–0.94) and the “low growth and extreme possibility” groups (OR = 0.83, *P*<0.01, 95%CI:0.79–0.87) demonstrated significantly lower levels of resilience compared to the high appreciation-power group.

**Conclusion:**

The PTG patterns in only-child-lost parents were varied. Promoting resilience may be a way to foster these parents’ PTG. Targeted intervention should be developed based on the characteristics of each latent class, and timely attention must be paid to the mental health of OCL parents who are without a spouse and have low income.

## Introduction

Since the early 1980s, the one-child policy has been implemented nationwide in China, leading to great changes in the Chinese population and family structure. With the increasing number of one-child families, a special group called the "only-child-lost family (OCL family)" has emerged. An OCL family is a family in which the parents have had only one child but now have no living biological or adopted children and in which the bereaved mother is older than 49 years (the maximum medical age for fertility in China) [1]. In China, the OCL family is also called the "Shi Du" family. OCL people are the bereaved parents in these families [2]. According to China’s health ministry and demographic estimates, the number of OCL families has increased to 10 million, with 20 million OCL people [3]. This large number indicates that OCL families have become a social phenomenon that cannot be ignored.

The majority of parents consider the death of a child the most serious incident in their lives [4]. Bereaved parents must address the rupture of a widely shared concept of what is perceived to be the natural course of life and are forced into reconstructing meaning [5]. This situation may cause severe and long-lasting grief [6]. Parents who have lost their only child experience a variety of emotional responses, such as anxiety, depression, risk of suicide, and prolonged pain [3], which may border on mental disorder [7].

In addition to the above emotional responses, posttraumatic growth (PTG) is also an issue for bereaved parents [5, 8, 9]. PTG is considered to be the “positive psychological change experienced as a result of the struggle with highly challenging life circumstances” [10]. PTG has been widely researched in the field of bereavement, including PTG in adolescents after the Sichuan Earthquake [11], couples 2–6 years after their premature baby died [12], bereaved university students [13], and bereaved parents [9].

Bogensperger and Lueger-Schuster found that 19 potentially beneficial themes emerged in bereaved parents' responses, among which the theme of personal improvement was most frequently cited (46.7%). Furthermore, responses concerning personal improvement were multifaceted, such as describing personal growth, having a greater trust in life, being more tolerant, or developing personal potential [5]. Additionally, different individuals may experience distinct patterns of PTG [14,15]. Thus, not everyone experiences growth in similar ways after a traumatic event [14]. There is heterogeneity in PTG among OCL people.

Currently, the main research methods for PTG involve computing a scale score to judge individuals’ PTG level. Cutoff points are used to judge whether a person has PTG, but different studies may use different cutoff points [11,16]. For example, according to Tang’s study, mean scores above 3 on the posttraumatic growth inventory were indicative of moderate levels of PTG in adult Thai survivors of the 2004 Southeast Asian earthquake-tsunami [16], whereas according to Yu, in adolescents after the Sichuan Earthquake, total scores above the 75th percentile were considered to have probable PTG [11]. It is obvious that the disparity in cutoff values could hinder the comparison of related studies' findings and the identification of precise recommendations for practice. Thus, we cannot use a total score to describe the state of PTG among OCL people.

Latent profile analysis (LPA) may be a suitable approach because it assumes that observed variables are indicators of an unobserved, latent variable, and it attempts to explain this relation in terms of a small number of subgroups or profiles [17]. Therefore, this method helps researchers to proceed with further analysis of subgroups’ characteristics and to obtain more in-depth results [18]. In addition, LPA is a person-centered analytic technique that derives profiles (i.e., subgroups) of individuals based on similar characteristic patterns (profiles) and differentiates homogeneous subgroups within a heterogeneous sample [19]. Therefore, it helps researchers to produce a probability estimation of all individuals belonging to a certain PTG group and finds the most rational classification according to the statistical indicators (e.g., fitting information). To our knowledge, no studies applying LPA have been conducted to explore subgroups of PTG among OCL people.

Another factor we examined was resilience. There is no general definition of this term accepted by scholars [20]. In this study, resilience is considered the ability to cope with and adapt to changes after threatening or challenging situations [21] because our aim was to explore how this ability affects traumatic outcomes and PTG. Resilience can help individuals to cope effectively with various adversities and can promote individuals’ recovery from negative events [22]. Previous studies of the relationship between resilience and PTG are inconsistent. One study found a positive correlation between resilience and PTG [23], since resilient people tend to adapt to changes in a cognitive process that resembles PTG [24]. Levine showed that high levels of resilience were associated with the lowest PTG scores [25]. This may be because resilient people tend to emerge from trauma relatively unchanged and with fewer psychological wounds, which makes these individuals less likely to perceive threats to their self or their world views and more likely to engage in meaning-making behavior [25,26]. However, growth requires an individual to find meaning for a traumatic event [27]. Previous studies of resilience and PTG have mostly concentrated on cancer patients [28], women with infertility [29], HIV patients [30], and people who have suffered from personal traumatic experiences in the previous year [31]. There is a lack of research on OCL people. Hence, it is necessary to test the resilience level of each PTG profile to examine whether resilient OCL people are more likely to report PTG.

The aim of this study was as follows: (a) to explore whether different profiles of PTG can be identified in OCL parents; (b) to determine the characteristics of each subgroup using a range of socio-demographic variables; and (c) to investigate the association between PTG and resilience. The findings of this study may provide a new perspective for developing psychological interventions and assistance mechanisms for OCL people.

## Methods

### Participants and procedure

The participants were bereaved parents who had lost their only child without a subsequent birth or adoption. The bereaved mothers were more than 49 years old, as defined by official documents. Individuals with serious mental disorders or physical illness who could not complete the questionnaires were excluded. The participants were residents of Changsha, the capital city of Hunan province in central China. Stratified random sampling was conducted by randomly selecting one sub-district from 9 administrative regions in Changsha. All OCL people registered in these sub-districts were included. The study was approved by the Institutional Review Board at the researchers’ university (grant number: 2015036). Data were collected between July 2014 and April 2015. Before the household visits, the researchers contacted community workers to provide lists of registered people and to obtain oral consent by telephone. Then, household visits were conducted by two trained research assistants. The anonymous questionnaires were issued after reconfirming the informed consent and were retrieved immediately when completed. For aged people with poor eyesight, the researchers read each item and completed it according to the participant’s answers. After finishing the questionnaires, a gift valued at 50 RMB (8 dollars) was provided to thank the participants for their cooperation.

### Measure

A questionnaire including 52 items was used in the study. The questionnaire consisted of the Post-Traumatic Growth Inventory, the Connor-Davidson Resilience Scale, and socio–demographic information. Approximately 10–25 minutes was needed to complete the questionnaire.

#### Post-Traumatic Growth Inventory (PTGI)

The PTGI was developed by Tedeschi in 1996 to assess a person’s growth after suffering a trauma, both physically and psychologically [32]. The PTGI is measured using a 6-point scale (0–5). Scores range from 0–105, with higher scores reflecting greater growth. The previous PTGI consisted of 21 items divided into 5 factors. The Chinese version of the PTGI was translated and revised in 2011 for accidental trauma patients older than 18 years, and 1 item was excluded due to cultural differences. The inventory was still divided into 5 factors: insights on life, personal power, new possibilities, relating to others and spiritual change [33]. The reliability coefficient of the entire scale and subscales in Chinese trauma patients was 0.661–0.846, and the Cronbach’s ἀ coefficient (ἀ) in this study was 0.909.

#### Connor-Davidson Resilience Scale (CD-RISC)

The CD-RISC was developed to assess psychological resilience among adults and is measured using a 5-point scale (0–4). Scores range from 0 to 100, with higher scores reflecting greater resilience. However, there are no specific demarcation points. The CD-RISC consists of 25 items divided into 5 factors [34]. The Chinese version of the CD-RISC was translated and revised in 2007 in a community population aged over 18 years old. The translated version still contained 25 items, but they were divided into 3 factors: tenacity, strength and optimism [35]. The reliability coefficient in the Chinese community was 0.91, and the Cronbach’s alpha coefficient (ἀ) in this study was 0.924.

#### Socio-demographics

Age, gender, place of residence, education level, per capita monthly income, diagnosed disease, and marital status were included to obtain the demographic characteristics of latent profiles of PTG.

### Statistical analysis

SPSS 18.0 and Mplus 7.0 (using an exploratory LPA) were used to analyze the data. First, a number of models, from the initial model (one profile) to the final one (6 profiles) were estimated by gradually increasing the profile number until the fit indices achieved the best fit. The relative fit indexes were evaluated, including Bayesian Information Criteria (BIC) and Adjusted Bayesian Information Criteria (aBIC), with smaller values indicating better model fit. LMRT (Lo-Mendell-Rubin Test) and BLRT (Bootstrap Likelihood Ratio Test) were considered as relative fit indices to determine whether the model was the best fit for the data. A probability value <0.05 indicated that the model fit the data significantly better compared to the previous one. In addition, when the LMRT and BLRT values become non-significant (>0.05), it indicated that the previous model was the best one [36]. Entropy was a marker of the clarity of class delineation, with values closer to 1 indicating fewer classification errors [37]. Generally speaking, if a model has higher entropy and lower BIC, and aBIC and achieves significant LMR and BLRT, the model has a greater fit [38].

Second, after choosing the optimal model, multinomial logistic regression was used to analyze the socio–demographic variables in each latent profile and the association between profile membership and resilience.

## Results

### Socio-demographic characteristics

A total of 249 questionnaires were issued and 222 questionnaires were returned, for an effective return ratio of 89.2%. The socio-demographic characteristics of the participants are provided in Table 1.

**Table 1:** Sociodemographic and clinical characteristics of the sample.

	n(%)/M±SD
Age	
<60	93(41.9%)
≥60	129(58.1%)
Gender	
Male	82(36.9%)
Female	140(63.1%)
Home Location	
Rural	34(15.3%)
Urban	188(84.7%)
Marital Status	
With a spouse	161(72.5%)
Without a spouse	61(27.5%)
Education Level	
Junior high school and below	113(50.9%)
Senior high school and above	109(49.1%)
Income per Month	
≤1000 ($156)	48(21.6%)
1001–2000 ($156-$312)	102(45.9%)
>2000 ($312)	72(32.4%)
Diagnosed Diseases	
With	98(44.1%)
Without	124(55.9%)
CD-RISC	56.90±16.92
PTG	53.13±13.38

Note CD-RISC: Connor-Davidson Resilience Scale, PTG: posttraumatic growth; Income is valued in RMB; 1000 RMB is worth approximately $156.

### Latent profiles of posttraumatic growth

A total of 6 models were estimated during the exploration. Each fit index is shown in Table 2.

**Table 2:** Fit indices of each model.

Model	K	BIC	aBIC	Entropy	LMR *P*	BLRT *P*
1	40	15962.88	15836.11			
2	61	15050.19	14856.87	0.932	0.00	<0.001
**3**	**82**	**14842.18**	**14582.31**	**0.919**	**0.02**	**<0.001**
4	103	14720.93	14394.52	0.943	0.51	<0.001
5	124	14688.46	14295.49	0.941	0.40	<0.001
6	145	14679.45	14219.93	0.943	0.53	<0.001

Note K = Number of free parameters; BIC = Bayesian information criterion; aBIC = sample size adjusted Bayesian information criterion; LMR = Lo-Mendell-Rubin likelihood ratio test; BLRT = parametric bootstrapped likelihood ratio test; *P* = probability.

As indicated by the fit indices, a three-profile solution provided the best theoretical and empirical fit to the data. Judging from the indices, the BIC and aBIC of 3 profiles were lower compared with 2 profiles and the LMR value (0.02) was significant in a model of 3 profiles, which meant that a 3-profile was better than a 2-profile model. The LMR value (0.51) was nonsignificant in a model of 4 profiles. Nylund & Muthén [39] suggested stopping the addition of profiles when the LMR becomes nonsignificant. The BLRT value was used to verify that the chosen model provided an appropriate fit to the data relative to a model with fewer profiles instead of identifying the best-fitting model because BLRT has been found to overestimate the number of profiles and the BLRT value did not contribute to distinguishing between models. Therefore, the three-profile model was considered optimal.

### Features and names of each latent class

**Fig 1:**
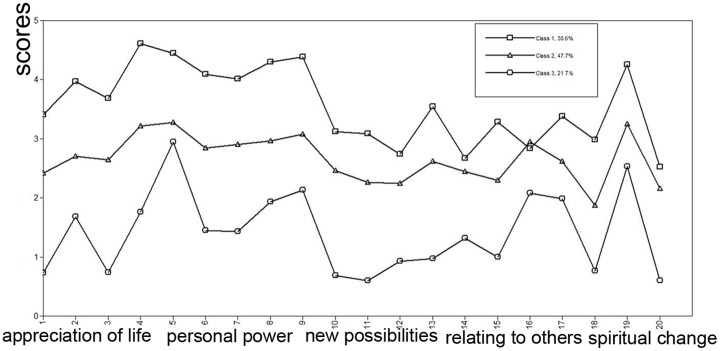
Latent profiles of PTG among OCL people. (A) For analytical purposes, we sort the items of C-PTGI by a factor that corresponds to 1–6 for appreciation of life, 7–9 for personal power, 10–13 for new possibilities, 14–16 for relating to others, 17–20 for spiritual change.

The scores of the four profiles on 20 items are presented in Fig 1, and each profile is named based on the probabilities of all items.

Profile 1 was described as the "high appreciation-power" group, which accounted for 30.6% (n = 68) of the sample. It was notable that this class showed the highest level of PTGI items of all profiles, especially in the appreciation of life and personal power factors. Thus, Profile 1 showed the greatest PTG, was much more effective at conquering adversity, and could better address difficulty.

The next group (Profile 2) was described as the "general moderate growth" group, which accounted for 47.7% (n = 106) of the sample. This group showed a moderate level of all items and a relatively stable growth trend.

The third profile (Profile 4) included the remaining 21.7% (n = 48) of the sample. This group reported the lowest levels of all items, especially in the factor of “new possibility”, and was described as the "low growth and extreme possibility" group.

Notably, 3 groups showed a peak on the graph for item 19 ("I have a greater feeling of self-reliance"). This finding may indicate that OCL people achieved a greater feeling of self-reliance.

### Predictor of latent profile membership

A multinomial logistic regression was conducted to explore the sociodemographic and clinical predictors of profile membership with the low growth and extreme possibility group as the reference group (see Table 3). Compared to those in the high appreciation-power group, individuals with monthly income >2000 ($312) were less likely to be in the general moderate growth group (OR = 0.13, *P*<0.01), and individuals with a spouse were less likely to be in the low growth and extreme possibility group (OR = 0.43, *P*<0.01).

**Table 3:** Predictor of latent profile membership.

	B	SE	Exp(B)	95% Confidence interval	*P*
P2 general moderate growth group (vs. P1 high appreciation-power group)					
Age<60	0.43	0.42	1.53	0.67–3.52	0.31
Gender, Ref.:female	0.29	0.42	1.33	0.56–3.18	0.52
Home Location, Ref.:urban	0.40	0.58	1.50	0.48–4.66	0.49
Marital status Ref.:without a spouse	-0.52	0.44	0.60	0.25–1.43	0.25
Education level, Ref.: senior high school and above	0.46	0.43	1.58	0.68–3.67	0.28
Monthly income:1001–2000	-0.91	0.55	0.40	0.14–1.17	0.10
Ref.: Monthly income:≤1000^a^					
**Monthly income:>2000**	**-2.02**	**0.62**	**0.13**	**0.04–0.45**	**<0.01**
**Ref.: Monthly income:≤1000**					
Diagnosed diseases, Ref.:With diseases	0.02	0.41	0.95	0.46–2.29	0.95
P3 low growth and extreme possibility group (vs. P1 high appreciation-power group)					
Age<60	0.24	0.35	1.27	0.64–2.53	0.49
Gender, Ref.: female	-0.04	0.36	0.96	0.48–1.93	0.91
Home Location, Ref.: urban	0.86	0.49	2.35	0.90–6.17	0.08
**Marital status Ref.: without a spouse**	**-0.84**	**0.37**	**0.43**	**0.21–0.90**	**0.02**
Education level, Ref.:senior high school and above	0.09	0.34	1.09	0.56–2.14	0.80
Monthly income:1001–2000	-0.22	0.50	0.81	0.31–2.13	0.67
Ref.:Monthly income:≤1000					
Monthly income:>2000	-0.65	0.51	0.52	0.19–1.41	0.20
Ref.:Monthly income:≤1000					
Diagnosed diseases, Ref.:With diseases	-0.58	0.33	0.56	0.29–1.07	0.08

Notes:

^a^: Income is valued in RMB; 1000RMB is worth approximately $156; Ref. = reference

### Relationship between the latent profiles of PTG and resilience

A multinomial logistic regression indicated that individuals in the "general moderate growth" group (B = -0.09, SE = 0.02, OR = 0.92,*P*<0.01,95%CI:0.89–0.94) and the "low growth and extreme possibility" group(B = -0.19, SE = 0.02, OR = 0.83,*P*<0.01,95%CI:0.79–0.87) demonstrated significantly lower levels of resilience compared to the "high appreciation-power" group.

## Discussion

### Latent profiles of PTG

This is the first known study to identify latent profiles of PTG among OCL people by LPA. This analysis differs from previous studies that have used different cutoff values. From the perspective of the practical classification technique, in contrast to cluster analysis, LPA has a lower classification for arbitrariness and provides more statistical functions, such as limited model parameters according to theory or practice [40]. Thus, LPA makes the classification more objective and realistic. From a person-centered perspective, this approach helps researchers to identify the different subgroups of PTG among individuals after an only child dies and to identify the socio-demographic characteristics of each group, which is useful for developing targeted psychological interventions. Previous interventions were mainly based on the individual scale score to determine the level of PTG, and included low scores in interventions. The internal heterogeneity of these people was ignored, such as different reactions by individuals to specific items. Furthermore, the population that obtained high scores were generally not considered for interventions; this situation overlooks low scores on some items by high-scoring individuals. The general implementation of the program has not been adjusted according to the characteristics of specific populations.

Consistent with a previous study on posttraumatic reactions [14], the findings of this study demonstrate that the PTG of OCL people has obvious features that contribute to classification and that support a three-profile model: a "high appreciation-power group", a "general moderate growth group", and a "low growth and extreme possibility group". This classification reflects the heterogeneity of OCL people in each latent profile of PTG.

The high appreciation-power group, which constituted 30.6% of individuals, had the highest level of PTG items among the profiles, especially in the appreciation of life and personal power factors. The individuals in this group have a greater ability than other profiles to cope with highly challenging life circumstances and perceive more positive life changes despite the greater distress associated with these tragic experiences. The PTG of bereavement was not identical for everyone. Some bereaved people show greater growth in particular aspects [41]. According to Dong and Chen, some OCL people are willing to participate in support groups to help other people by playing the role of rights defenders and organizers [42]. A number of studies have suggested that peer support is an effective intervention for bereaved people [43–46]. The individuals in this group may play an important role for their peers. Social workers should encourage these individuals to devote themselves to helping their peers.

The low growth and extreme possibility group, which constituted 21.7% of individuals, had the lowest levels of all items, especially in the “new possibility” factor. In this group, OCL people do not want excessive contact with the outside world to seek new possibilities. In the past, we may have ignored this special requirements for intervention with this group. However, it is important to pay attention to this group and strengthen their contact with others to help them develop new interests. According to The Alliance of Perinatal Bereavement Support Facilitators, maintaining individuals’ interest and involvement is a critical factor in developing effective communication and forming a collaborative, supportive atmosphere among all members [47, 48]. Thus, social workers should consider OCL individuals’ interests and should provide opportunities to engage in collective discussion and problem solving.

Interestingly, this study showed a high score on the graph for item 19 ("I have a greater feeling of self-reliance"), which might be explained in the following way. On the one hand, Chinese traditional culture dictates that the purpose of children is to look after their parents in their old age. When people lose their only child, they can no longer rely on their child and must rely on themselves when they are older. On the other hand, traumatic experiences can increase individuals’ self-protective function and enhance their courage to face the future, thereby providing a positive experience [49].

### Socio-demographic characteristics of each latent profile

The results indicate that demographic factors, such as age, gender, home location, with or without diagnosed disease, and education level, did not differ significantly among these 4 profiles. The findings were inconsistent with some prior studies [50, 51]. The difference may be due to the cultural influence, which was considered a crucial factor in adaptation following the death of a child [52]. In addition, based on Bowlby's attachment theory, different attachment styles are related to different levels of psychological reactions [53]. An only child has care and love from the entire family, and the attachment in this type of family would be close. As suggested in a previous qualitative study [6], the death of an only child is an event that produces “deep sorrow” and “a heavy blow”. In summary, there were no significant differences in these common demographic variables.

The results of this study indicated that OCL people with a spouse were more likely to be in the high appreciation-power group. Individuals who deal with a traumatic experience such as losing an only child may recognize their vulnerability and be willing to accept help [54]. In this case, there would be a committed partner in a stable family who can offer beneficial support to cope with or improve the individual’s situation. There was a significant difference in per capita monthly income. To a certain extent, increased income would contribute to higher PTG for OCL individuals. The stress of everyday life resulting from the death of a child continuously affects individuals, which may further limit their PTG. This finding is consistent with Wang’s assertion that economic factors can help to alleviate the daily pressures of individuals and produce a higher level of PTG [55].

### Resilience of three profiles

In this study, individuals in the general moderate growth group and the low growth and extreme possibility group demonstrated significantly lower levels of resilience compared to the high appreciation-power group. Some researchers suggest that people with higher resilience have much more cognitive flexibility. When faced with a traumatic event, they can be more flexible and gain PTG [10]. This assertion is supported by the Model for Personal Growth and Relationships (OTHERS), which is summarized in a study on bereavement [56, 57]. This model notes that resilience is one of eight core resources that help individuals improve PTG. Our findings indicate that higher resilience may help individuals gain more PTG. This finding is consistent with studies on bereavement, such as studies of spousal bereavement in old age [58], bombing survivors [59], and widows [60]. The results show that resilient bereaved individuals have positive outcomes. This finding may indicate that promoting resilience can be a way to foster PTG.

### Recommendations for policy-making

In recent years, the Chinese government has paid attention to this vulnerable group and has issued a series of policies to help them due to constant appeals. A nationwide policy provides a monthly financial aid grant depending on the economic level of the local government, ranging from 200 RMB to 800 RMB ($31.20-$124.80). In Changsha, where the samples were collected, OCL people were granted 400 RMB ($62.40) per month. However, OCL people were often an old, unemployed, low-income group, and this financial aid grant cannot guarantee a minimum subsistence level. Our study results showed that high income (>$156) OCL people were more likely to be in the “high appreciation-power group”. Therefore, an elastic policy of financial aid to care for low-income bereaved parents should be established.

### Conclusions and limitations

In summary, obvious classification features for PTG emerged in this study, and 3 profiles were supported: a “high appreciation-power group”, a “general moderate growth group”, and a “low growth and extreme possibility" group”. Promoting resilience can be a way to foster PTG. Targeted interventions should be developed based on characteristics of each latent profile, and timely attention should be paid to the mental health of OCL people who are without a spouse or have low income.

Several limitations of this study need to be acknowledged. First, the study was cross-sectional, so the causation between resilience and PTG cannot be extrapolated. It is recommended that growth should be studied longitudinally [61]. Thus, future research should track PTG and examine risks as well as protective factors related to PTG over time. Second, although the relationship between the gender, cause of death, and age at death of the child have been examined in many studies, this study did not investigate these factors due to ethical considerations. In our future works, we will identify trends and develop an appropriate intervention program to conduct further empirical research.

## Supporting Information

S1 Data (XLS)
